# Evaluation of the Risk of Urinary System Stone Recurrence Using Anthropometric Measurements and Lifestyle Behaviors in a Developed Artificial Intelligence Model

**DOI:** 10.3390/diagnostics15202643

**Published:** 2025-10-20

**Authors:** Hikmet Yasar, Kadir Yildirim, Mucahit Karaduman, Bayram Kolcu, Mehmet Ezer, Ferhat Yakup Suceken, Fatih Bicaklioğlu, Mehmet Erhan Aydin, Coskun Kaya, Muhammed Yildirim, Kemal Sarica

**Affiliations:** 1 Department of Urology, Sancaktepe I.Varank Training and Research Hospital, 34785 Istanbul, Turkey; yasarhikmet193@gmail.com (H.Y.); kemal.sarica@sbu.edu.tr (K.S.); 2 Department of Urology, Elazig Fethi Sekin City Hospital, 23280 Elazıg, Turkey; kadir.yildirim@sbu.edu.tr (K.Y.); bayramkolcu@hotmail.com (B.K.); 3 Department of Software Engineering, Malatya Turgut Ozal University, 44100 Malatya, Turkey; mucahit.karaduman@ozal.edu.tr; 4 Department of Urology, Medical School, Kafkas University, 36040 Kars, Turkey; mehmetezer@gmail.com; 5 Department of Urology, Umraniye Training and Research Hospital, 34764 Istanbul, Turkey; ykpsckn@gmail.com; 6 Department of Urology, Kartal Lutfi Kirdar Training and Research City Hospital, 34865 Istanbul, Turkey; fatihbicaklioglu@hotmail.com; 7 Department of Urology, City Training and Educational Hospital, 26080 Eskisehir, Turkey; merhanaydin@gmail.com (M.E.A.); coskun.kaya@sbu.edu.tr (C.K.); 8 Department of Computer Engineering, Malatya Turgut Ozal University, 44000 Malatya, Turkey; 9 Department of Urology, Medical School, Biruni University, 34015, Istanbul, Turkey

**Keywords:** autoencoder, artificial intelligence, clinical decision support system, stone recurrence, urinary system stone disease

## Abstract

**Background/Objectives:** Urinary system stone disease is an important health problem both clinically and economically due to its high recurrence rates. In this study, an innovative hybrid approach based on deep learning is proposed to predict the recurrence risk of stone disease. **Methods:** Patient data were divided into three subsets: anthropometric measurements (Part A), derived body composition indices (Part B), and other clinical and demographic information (Part C). Each data subset was processed with autoencoder models, and low-dimensional, meaningful features were extracted. The obtained features were combined, and the classification process was performed using four different machine learning algorithms: Extreme Gradient Boosting (XGBoost), Cubic Support Vector Machines (Cubic SVM), k-Nearest Neighbor algorithm (KNN), and Decision Tree (DT). **Results:** According to the experimental results, the highest classification performance was obtained with the XGBoost algorithm. The suggested approach adds to the literature by offering a novel solution that makes early risk calculation for stone disease recurrence easier. It also shows how well structural feature engineering and deep representation can be integrated in clinical prediction issues. **Conclusions:** Prediction of the stone recurrence risk in advance is of great importance both in terms of improving the quality of life of patients and reducing the unnecessary diagnostic evaluations along with lowering treatment costs.

## 1. Introduction

The prevalence of urinary system stone disease is increasing worldwide. Although the incidence of stone disease was reported as 6.5% in the United States in 2008, it increased to 10.9% by 2018 [1]. Urinary system stone disease is the reason why millions of people visit the emergency department every year [1,2]. The person usually describes pain caused by urinary system stone disease as the worst pain experience in their life. Along with the decrease in quality of life originating from the severe pain at every attack and the relevant problems, this situation leads to social and economic problems [2]. The recurrence rate of kidney stones is quite high; in the literature, recurrent stone formation has been reported in approximately 50% of patients within 10 years after the first stone episode [3]. Recurrence is a significant problem that negatively impacts the quality of life for patients and imposes a substantial economic burden on the healthcare system. Factors affecting the recurrence of kidney stones include a family history of stones, the presence of stones at an early age, dietary habits, the chemical composition of the stone, metabolic disorders, environmental factors, lifestyle, and the presence of an infection [1,4]. Metabolic abnormalities such as hypercalciuria, hypocitraturia, hyperuricosis, and inadequate fluid intake have been identified as the main factors that increase the risk of recurrence [5].

The risk of recurrence necessitates the long-term follow-up of patients and implementation of individualized preventive strategies. However, the early and reliable prediction of recurrence risk with traditional methods is often limited; clinical decisions are usually based on physician experience and limited biomarkers. In recent years, artificial intelligence and machine learning techniques have become promising tools for predicting stone recurrence by extracting meaningful patterns from large and multidimensional patient data. Artificial intelligence-based representation learning methods, in particular, enable high-accuracy inferences to be made from both clinical and biometric data due to their capacity to model complex relationships. The effective use of these technologies in the healthcare system can detect the risk of recurrence in kidney stones at an early stage. Thus, personalized treatment plans can be developed and patient outcomes can be improved. This can also enable the more effective use of existing resources.

Although some factors affecting the recurrence of kidney stones have been identified, it is not precisely known in which patients it will recur and in which patients it will not. The model developed in this study aims to predict the risk of recurrence by entering patient data, thus contributing to the individualization of treatment. Today, the use of artificial intelligence as a decision support system in healthcare, particularly in the stages of diagnosis, treatment, and disease prediction, is becoming increasingly widespread. Especially in the analysis of large datasets, thanks to the learning ability and fast evaluation features of artificial intelligence, it can reveal more complex relationships than classical statistical methods and produce valuable results. In this case, where recurrence risk prediction is clinically challenging, the fact that it analyzes patient characteristics reveals relationships and makes predictions which reveals the importance of artificial intelligence in this field. Thanks to this feature, determining patients at risk of kidney stone recurrence in advance will be beneficial both in terms of patient comfort and in planning and implementing preventive treatment.

This study presents a novel approach based on a multidimensional representation of patient data for predicting the recurrence risk of urinary stone disease. While raw data is typically provided directly to classification algorithms in the literature, in this study, the data were divided into three structural groups: Part A, Part B, and Part C. Low-dimensional but information-rich features were obtained by using separate autoencoder architectures for each group. The integrated feature set created by combining these representations was evaluated using powerful classification algorithms, including XGBoost, Cubic SVM, Fine KNN, and Decision Tree (DT). Notably, the high accuracy rates obtained with the XGBoost classifier suggest that the proposed approach can be an effective and reliable tool for predicting stone recurrence. In this respect, the study makes an original contribution to the literature by presenting a rare example specific to stone disease based on the hybrid use of deep learning-based representation and classical machine learning classifiers.

The rest of the paper is divided into four sections. In the Materials and Methods section, the models and classifiers used in the study are examined, and the proposed model and dataset are presented. The Experimental Results section, which follows, includes experimental results and the evaluations derived from these results. In the Discussion section, detailed discussion, comparisons, and limitations of the study are being discussed. The relevant paper is concluded with the Conclusions section.

## 2. Materials and Methods

**Figure 1:**
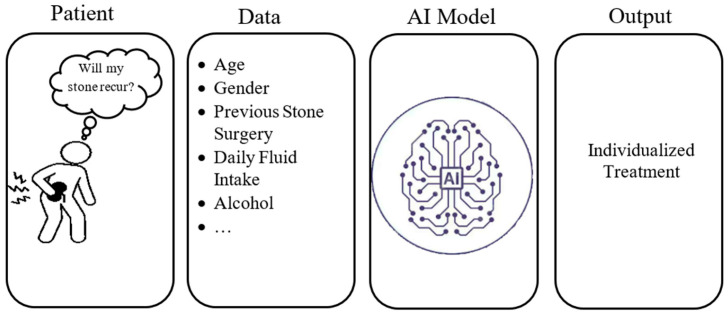
Incoming flow chart of the study.

The block diagram of this study performed to predict stone recurrence is roughly presented in Figure 1.

### 2.1. Dataset

This study was initiated after receiving ethical approval from the Eskişehir City Hospital Ethics Committee. 562 patients from different regions of Turkey, including Elazığ Fethi Sekin City Hospital (95), Eskişehir City Hospital (87), Sancaktepe City Prof. Dr. İlhan Varank EAH traning and research hospital (44), Kafkas University (139), Kartal Education and Research Hospital (124), Ümraniye Education and Research Hospital (73), were included in the study. While creating the dataset, the data obtained from 562 patients are listed as Age, Gender, Education Status, Diabetes, Hypertension, Cardiovascular Disease, Inflammatory Bowel Disease, History of Intestinal Surgery, Family History of Stone Disease, Daily Water Consumption, Daily Movement, Tea Consumption, Coffee Consumption, Salt Consumption, Animal Protein Consumption, Living Climate, Occupation Type, Use of Drugs Associated with Stone Formation, Sweating, Smoking, Alcohol, Height (cm), Weight (kg), Waist Circumference (Wm), Hip Circumference (hm), BKI, Body Shape Index Bsi), Body Roundness Index (Bri), Waist-Height Ratio (Wthr), Taper Index (Ci), Waist-Hip Ratio (Whr), and Stone Disease, and the relevant answers are recorded for each condition. The output data is whether there is a recurrence or not.

**Figure 2:**
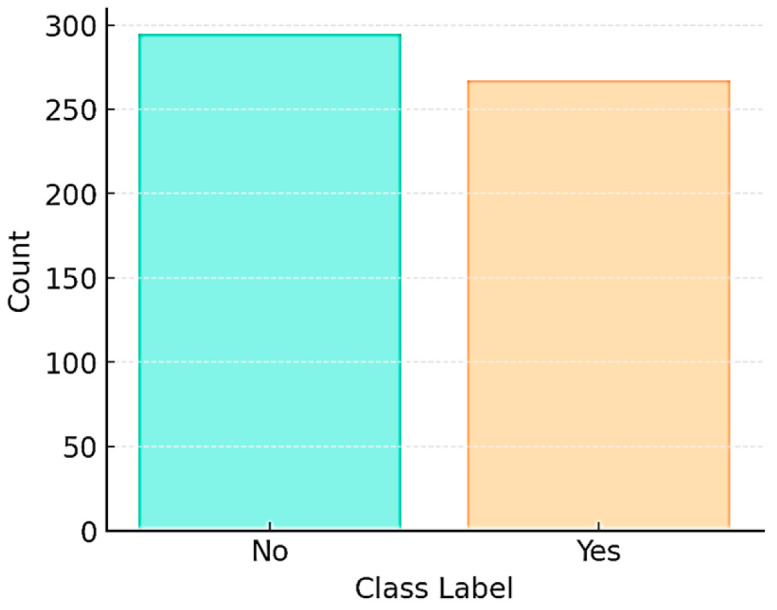
Class distribution of the dataset.

The class distribution of the examples in the datasets is shown in Figure 2.

There are 295 examples in the “No” class and 267 in the “Yes” class. These values indicate that the datasets have a generally balanced structure. The features in the dataset are presented in Table 1.

**Figure 3:**
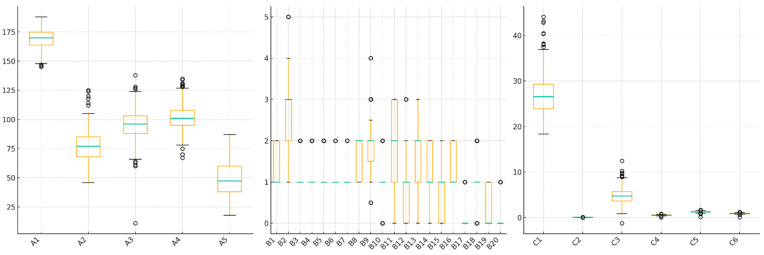
Feature Distributions of the Dataset.

Feature Distributions of dataset are presented in Figure 3.

**Figure 4:**
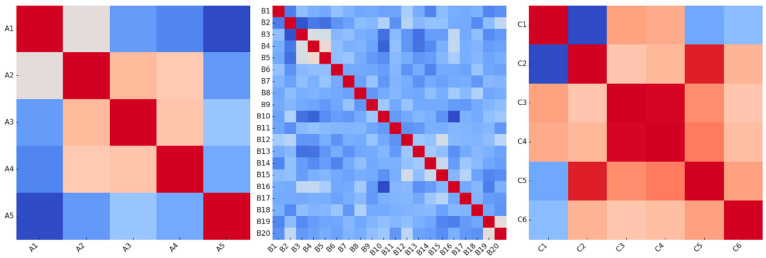
Correlation Heatmaps of Feature Relationships Across Dataset.

Box plots across the three parts show that the features have generally similar variance structures. While some features exhibit broader distribution in parts A and B, parts C exhibit narrower distribution ranges. Several features in part B exhibit significant outliers. Overall, the features are statistically balanced; however, some variables exhibit distinct variation in classification. Correlation Heat Maps of Feature Relationships Between parts are presented in Figure 4.

Correlation heatmaps indicate moderate positive correlations between attributes in parts A and B, while high correlations are observed between variables in part C. The strong correlation, particularly between A3–A4 (waist and hip circumferences), in part A indicates redundancy of information based on natural anthropometric relationships. Part B contains block structures where some lifestyle variables co-vary. In contrast, the derived body composition indices (e.g., BMI, BRI, WHtR) in part C are highly correlated due to their formulaic structure. Therefore, the variables in this section carry less independent information. Therefore, the contribution of parts A and B may be more decisive for generalization during the model development phase.

### 2.2. Autoencoder-Based Feature Extraction and Classifiers

Autoencoder structures were used as the basis for the model developed to predict the risk of recurrence of stone disease. In this study, to transform high-dimensional input data into meaningful representations, features are extracted using an autoencoder architecture [6]. Autoencoders are also utilized for feature extraction due to their ability to capture both linear and nonlinear relationships within the data. In this study, a structure consisting of an input layer and an encoding hidden layer is used. First, the model is trained, followed by the feature extraction process. The data in the dataset, divided into three parts, is given to the input separately, and features are extracted from each part. Then, the obtained features are concatenated to form a new feature vector.

The encoder and decoder stages for the autoencoder are given in Equations (1) and (2).

(1) $$z=f_{0}\left(\tilde{x}\right)=A\left(W_{e}\tilde{x}+b_{e}\right)\;\in \;R^{m}\;,\;\;A\left(u\right)=\max\left(0,u\right)(ReLU)$$

$W_{e}$ is the weight matrix of the encoder, $b_{e}$ represents the bias vector of the encoder, $A\left(u\right)$ is the activation function, $z\;\in \;R^{m}$ is the embedding vector and m = 8 is chosen.

(2) $$\hat{x}=g_{\theta }\left(z\right)=W_{d}z+b_{d}\;\;\;\in \;R^{d}$$

$W_{d}$ is the weight metric of the solver, $b_{d}$ represents the solver’s bias vector, and $\hat{x}$ are the structured input values generated by the solver. The loss function is calculated using Equation (3).

(3) $$\;\;L_{AE}\left(\theta \right)=\frac{1}{N}\sum_{i=1}^{N}{\left\|{\tilde{x}}^{i}-{\hat{x}}^{i}\right\|}_{2}^{2}$$

*N* represents the total number of samples, ${\tilde{x}}^{i}$ is the ith normalized real input vector, ${\hat{x}}^{i}$ is the input constructed by Autoencoder, $L_{AE}\left(\theta \right)$ is the mean square error value.

The parameters that constitute θ, which represents the parameter set, are ${\{W}_{e},\;b_{e},\;W_{d},\;b_{d})$ and are updated using the Adam optimization algorithm.

After this stage, the created feature vector is classified with XGBoost [7], Cubic SVM [8], Fine KNN [9], and Decision Trees (DT) [10] classifiers, and their performances are compared. The first classifier used for feature classification in the study is XGBoost. Extreme Gradient Boosting (XGBoost) is one of the most frequently used classifiers in supervised learning problems. This classifier is an ensemble classification method that produces successful results. XGBoost minimizes the error at each iteration. It also accelerates the optimization process by utilizing second-order derivatives of the loss function. It also employs L1 (Lasso) and L2 (Ridge) regularization methods in conjunction to prevent overfitting. XGBoost demonstrates successful classification performance on incomplete datasets thanks to its parallel computation and scalable structure [7].

Another classifier used in this study is SVM. SVM is another frequently preferred classifier for linear and nonlinear classification problems. As a supervised learning algorithm, SVM produces successful results in both linear and nonlinear classification problems. SVM separates samples with an optimal separating hyperplane that maximizes the margin. This maximizes generalization across classes and reduces the risk of overfitting the model. In nonlinearly separable cases, the data space is transformed into a higher-dimensional feature space using kernel functions. Cubic SVM is used in this study. It uses a third-order version of the polynomial kernel function. This kernel offers more sophisticated models of nonlinear decision boundaries, resulting in improved outcomes in high-dimensional datasets with inter-class overlap [8].

Another classifier used to predict the risk of recurrence of stone disease is KNN. KNN is another example-based supervised learning method. KNN performs classification using the labels of the nearest neighbors of the test sample in the feature space. KNN employs a similarity-based decision mechanism that utilizes various distance measurement metrics during the prediction phase. The value of k has a significant impact on the model’s performance. Low k values give the model high sensitivity, while high k values provide more robust generalization against noise. Fine KNN was used in this study. Fine KNN is a highly discriminative version of the classical KNN method. Fine KNN is particularly preferred for datasets with fine-grained class boundaries. This classifier sharpens the decision boundaries, enabling high-resolution discrimination. Fine KNN is frequently used in small or medium-sized datasets due to its parameter-free structure, low computational cost, and interpretability [9].

The final classifier used in this study to predict the risk of stone disease recurrence is the DT. DTs are one of the supervised learning techniques frequently used in both classification and regression problems. The high explainability of DTs allows for their frequent use. The DT algorithm sequentially partitions the dataset into branches based on the attribute values of each branch. This algorithm aims to obtain homogeneous classes at each leaf node. DT offers advantages such as modeling nonlinear relationships, handling missing data, and straightforward interpretation of results. However, because DRs are prone to overfitting, it is important to balance them with pruning techniques [10].

### 2.3. Proposed Model

**Figure 5:**
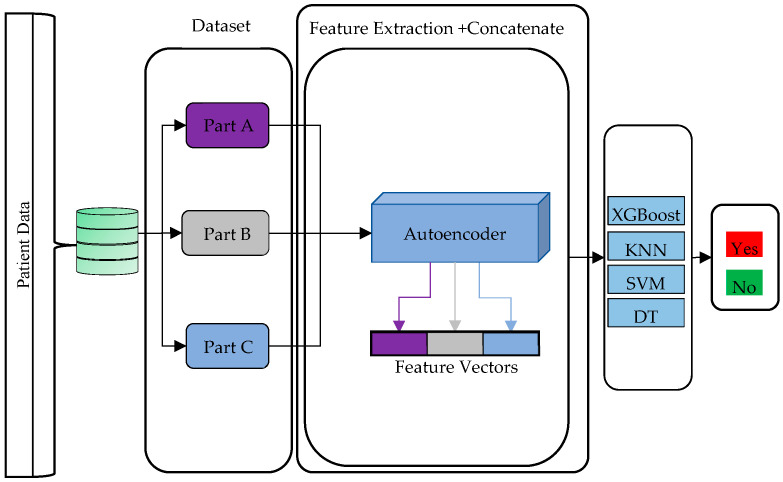
Proposed method.

In this study, a hybrid model was developed to estimate the risk of recurrence of stone disease. The data obtained from the patient dataset was first divided into three subcomponents. In this separation, Part A consists of the patient’s basic anthropometric features, including age, height, weight, waist circumference, and hip circumference. Part B includes derived body composition indicators such as Body Mass Index (BMI), Body Shape Index (ABSI), Body Roundness Index (BRI), Waist-to-Height Ratio (WHtR), Taper Index (CI), and Waist-to-Hip Ratio (WHR). The remaining clinical and demographic data were collected under Part C. In this study, each part of the dataset was divided into three separate parts and fed as input to a distinct autoencoder model. For each subdataset, low-dimensional and meaningful representations (features) were extracted by using the autoencoder architecture. Autoencoders can produce more representative features that are purified from noise by learning the complex and hidden relationships in high-dimensional data. In this way, the information extracted from different types of data was represented in a lossless manner. By combining these features, a more sensitive and accurate classification of stone recurrence was achieved. In the final stage, the combined feature set was evaluated with four different machine learning algorithms, XGBoost, Cubic SVM, Fine KNN, and DT classifiers. According to the experimental results, the highest accuracy and classification performance were obtained with the XGBoost algorithm. The general structure of the proposed model is given in Figure 5.

Patient data are provided as input to the model in three parts. The data matrices comprising these parts are denoted as $X^{\left(a\right)}\in \;R^{N\times d_{a}}$, $X^{\left(b\right)}\in \;R^{N\times d_{b}}$ ve $X^{\left(c\right)}\in \;R^{N\times d_{c}}$. The target column is $y\in \;{\left\{1,2\right\}}^{N}$ and operations are performed by applying $\tilde{y}=y-1\in \;{\left\{0,1\right\}}^{N}$ transformation for binary classification.

Each feature matrix is standardized column-wise before autoencoder training. Equations (4)–(6) are applied separately for each file.

(4) $$\mu_{j}=\frac{1}{N}\sum_{i=1}^{N}x_{ij}$$

$\mu_{j}$ j represents the arithmetic mean of all values in the jth column, $x_{ij}$ represents the j. feature of the i. sample in the data matrix, and N is the total number of samples in the dataset.

(5) $$\sigma_{j}=\sqrt{\frac{1}{N}\sum_{i=1}^{N}{{(x}_{ij}-\mu_{j})}^{2}}$$

$\sigma_{j}$ represents the feature standard deviation.

(6) $$\tilde{x_{ij}}=\frac{x_{ij}-\mu_{j}}{\sigma_{j}}$$

$\tilde{x_{ij}}$ is the transformed value obtained after Z-score standardization.

The performance of the proposed model was tested on different classifiers, both with and without autoencoder.

## 3. Results

The performance of the proposed model and the performance of the models used for comparison were evaluated using different metrics. Accuracy, precision, recall, and F1-score metrics are calculated to compare the performance of the classifiers. Performance metrics are calculated with accuracy (Equation (7)), precision (Equation (8)), recall (Equation (9)), and F1 score (Equation (10)). In the equations, True Positive value is expressed as TP, True Negative value as TN, False Positive value as FP, and False Negative value as FN.

(7) $$Accuracy=\frac{TP+TN}{TP+TN+FP+FN}$$

(8) $$Precision=\frac{TP}{TP+FP}$$

(9) $$Recall=\frac{TP}{TP+FN}$$

(10) $$F1-Score=2\cdot \frac{Precision.Recall}{Precision+Recall}$$

Each of these performance metrics is used to evaluate the model’s performance from different aspects. Thus, it is possible to make accurate analyses of model performance. Accuracy performance metric determines the correct prediction rates of the model among all predictions and is used to measure the overall success of the model. Precision indicates the accuracy of the data predicted as positive in the prediction results, specifically the proportion of truly positive cases. It is used in cases where the false positive rate is important and is useful in reducing the risk of unnecessary treatment in clinical practice. Recall is a performance metric used to determine how many of the true positive examples are predicted correctly. It is used so that true positives are not overlooked in the study. It is desired that the recall value is high to prevent false negatives from yielding negative results. Finally, the F1-score metric is calculated using the harmonic mean of the precision and recall metrics. It provides a performance measurement by establishing a balance between these two values in case of unbalanced data. Thanks to these metrics, the overall performance of each model is measured, and multi-faceted analysis is performed, thus providing benefits in terms of increasing the reliability of decision support systems.

In the dataset, 31 different values obtained from 562 people were divided into three parts, labeled A, B, and C, and given to the autoencoder. The parts were used separately to extract features. The features extracted from the autoencoder were combined and divided into 80% training and 20% test. Models were trained and evaluated using a stratified 80/20 train-test split with a fixed random state to ensure repeatability. No k-fold cross-validation or repeated random sampling was applied in this study. The performance results obtained as a result of the classification of the features taken in the part of the dataset where recurrence was checked are given in Table 2. While the performance comparison was made for XGBosst, Cubic SVM, Fine KNN, and DT, the performance metrics compared are accuracy, precision, recall, and F1-score values.

**Figure 6:**
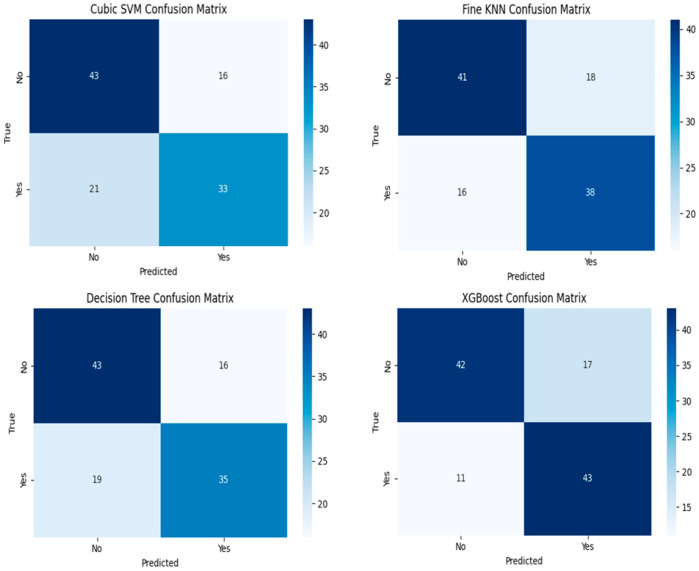
Classifiers with autoencoder results confusion matrix.

The proposed model achieved the highest performance with 75.22% in the XGBoost classifier. While the accuracy value achieved by XGBoost in the Recurrence (−) class was 71.18%, this value was 79.62% in the Recurrence (+) class. Among all the tested classifier models, the XGBoost model proves to be the most suitable for classifying data on whether there is a recurrence. The balanced precision, recall, and F1-score rates indicate that the model is strong in making correct predictions and is successful in detecting recurrence cases. DT made a correct prediction with a performance score of 72.57%. While the accuracy value obtained by DT in the Recurrence (−) class is 71.18%, this value is 74.07% in the Recurrence (+) class. The Fine KNN classifier results in a threshold value of 70%, but this neighborhood-based approach can lead to time and performance losses in extensive datasets. Cubic SVM is the classifier with the lowest performance. While the accuracy value obtained by Cubic SVM in the Recurrence (−) class is 72.88%, this value is 61.11% in the Recurrence (+) class. This indicates that the number of erroneous predictions is higher compared to other models, and it is less likely to be used as a result. The confusion matrix for the classifiers, which shows the best performance for data on whether there is a recurrence, is given in Figure 6.

When the confusion matrices in Figure 6 are examined, the most successful classifier is XGBoost. When the performance analysis of the confusion matrix is evaluated, it is seen that 42 out of 59 patients who did not have a recurrence were predicted correctly. Additionally, it was observed that 17 patients were incorrectly identified and subsequently decided to undergo a recurrence. While the model correctly predicted 43 out of 54 patients who had a recurrence, it incorrectly predicted 11 as having no recurrence. This situation demonstrates that the model yields successful results in predicting whether patients will experience a recurrence, based on the data. The overall performance of the classifier with and without autoencoder features is compared in Table 3.

**Figure 7:**
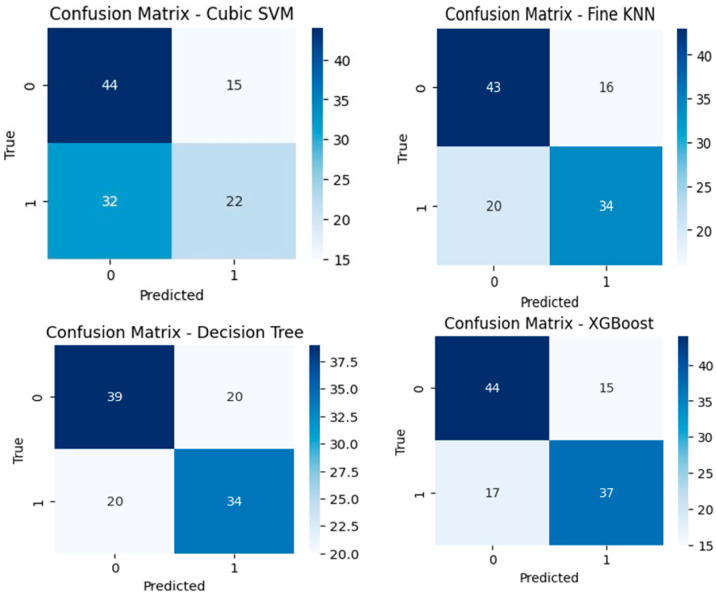
Classifiers without autoencoder results confusion matrix.

Confusion matrices obtained without using autoencoder are presented in Figure 7.

When Figure 7 is examined, it is observed that the most successful classifier is XGBoost. Evaluating the confusion matrix of the XGBoost classifier revealed that 44 of 59 patients were correctly predicted as recurrence (−). Furthermore, 15 patients were incorrectly identified as recurrence (+). The model correctly predicted 37 of 54 patients as recurrence (+), while incorrectly predicting 17 as recurrence (−). This classifier correctly predicted 81 data and incorrectly predicted 32 data. As a result, the XGBoost classifier produced the most successful results both when the autoencoder was used and when it was not used. In both cases, the worst performance is obtained in the Cubic SVM classifier.

## 4. Discussion

Kidney stone disease is a significant urological issue that is increasing in prevalence worldwide and tends to recur frequently. It has been reported in the literature that recurrence develops in approximately half of the patients within 10 years after the first stone formation. This situation necessitates that kidney stones be treated as a chronic condition rather than merely an acute issue. Stone recurrence not only negatively affects the quality of life of the patients but also imposes severe economic burdens on the health system due to repeated emergency visits, surgical interventions, and imaging procedures. Therefore, it is of great clinical importance to perform an early risk assessment of stone patients in terms of recurrence and to plan preventive approaches accordingly.

In this study, an artificial intelligence model was developed to predict the recurrence of kidney stones in patients who had previously undergone surgery for kidney stones. Patient history and laboratory data were evaluated and patients who were likely to have stone recurrence were successfully predicted with high accuracy and sensitivity. Thus, it is promising for the implementation of individualized treatment and follow-up options for patients with kidney stones.

Although the prevalence of kidney stones in society is reported to be approximately 10%, this rate may vary depending on the specific society. This rate can reach 20% in Middle Eastern and Asian societies. In patients who have previously undergone surgery for kidney stones, stone recurrence has been reported as approximately 50% within 10 years [11]. In the study, it was reported that stone recurrence rates were 30–40% within 5 years and 10–15% in the first year after surgery [11]. Additionally, it is predicted that stone prevalence and recurrence rates will increase further due to global warming and climate change [12]. Such high rates of kidney stone recurrence pose a significant economic burden for both individuals and healthcare systems. In a study conducted by Elias et al., it was stated that urinary system stone disease directly exceeds approximately USD 5 billion per year in costs to the US health system [13]. Although it is predicted that recurrence may be more likely in patients with certain risk factors (family history, infection, genetic stone disease, dietary habits, patients with multiple stone recurrences, obesity, fluid intake, etc.), it is not clear which patients will recur and which will not. Potential advantages of our model in clinical practice include its ability to facilitate risk assessment and, consequently, patient-centered treatment plans.

Several essential studies in the literature have been conducted on stone detection from images, while others have examined the factors that affect its formation. There are numerous studies in the literature that utilize Computerized Tomography (CT) images for detection. Various methods, including image processing techniques, deep learning algorithms, classical segmentation algorithms, and spectral analysis, are employed in studies conducted using this method. There are studies in the literature for the Prediction of Recurrence risk in urinary system stone disease. However, these studies generally evaluate a few metrics. In our study, a comprehensive evaluation was conducted using artificial intelligence, encompassing different metrics. In addition, most of these studies in the literature tried to determine whether there was a stone or not. The number of studies conducted to predict stone recurrence is relatively low. Therefore, the accuracy value obtained in this study is very important. Skolarikos et al. made a comprehensive review with a limited number of metrics in their study. They stated that patients should be given general instructions on how to prevent recurrence, including adequate fluid intake, calcium intake, low sodium consumption, and protein consumption. It has been stated that identifying and correcting causal factors is an essential tool in preventing the recurrence of urolithiasis [14].

Guo et al. stated that urinary stone recurrence significantly increases resource consumption and socioeconomic burdens in healthcare services. They stated that the underlying reasons for this are still unclear and that it is essential to determine critical risk factors to determine these reasons. In the study, these risks were determined with methods based on machine learning. In this study, 1416 patients with urinary stones were analyzed. In the study, an AUC value of 0.74 was obtained using random forest [15]. Mahmudi et al. used five different machine learning methods in their study to investigate the factors associated with kidney stone disease. It was stated that 10,128 participants were included in the study. As a result of the experimental studies, it was stated that the highest AUC value of 0.60 was obtained in the XGB model [16]. Lee et al. discussed the relationship between inflammation markers and kidney stones. In this study, it was stated that nephrolithiasis has a worldwide prevalence of 9% and a recurrence rate of nearly 50%. In this study, it was also noted that urinary stones have a significant impact on quality of life and pose a substantial burden on healthcare systems. Systemic inflammation is considered a risk factor for urinary stones. Previous studies have found a relationship between inflammatory markers and kidney stones; however, these findings are often based on patient recall, which introduces potential recall bias. This study investigates whether inflammatory markers vary according to the presence of nephrolithiasis using health check-up data from a large cohort in South Korea [17]. Eyre et al. investigated the clinical value of blood tests in screening for metabolic disorders in patients with kidney stones. Biochemical data collected from 709 patients were analyzed, and it was found that only serum calcium measurement was effective in identifying clinically significant conditions such as primary hyperparathyroidism. They recommended a 24 h urine analysis for patients with recurrent stones to obtain a reliable result [18]. Zhu et al. developed an LSTM-based deep learning model based on blood and urine test data to detect kidney stones at an early stage. Retrospective data obtained from 1130 patients and 1230 healthy individuals were used for training and testing in the model. The four most critical predictive variables were urine WBC, occult blood, protein presence, and microset percentage, indicating that routine laboratory parameters could be valuable in predicting stone disease [19]. Jiang et al. used clinical features and a nomogram model to estimate the risk of recurrence of complicated urinary tract infection in pediatric patients in their study. Data from 421 pediatric patients were used in the study. Recurrence was repeated in 288 patients at a rate of 68.4% during the 22.9-month follow-up period. A logistic regression model was used for the predictions. In the study, it was stated that complicated urinary tract infections (cUTIs) have a higher incidence of antibiotic resistance, recurrence, chronicity, and progression [20].

It is known that metabolic, genetic, and environmental factors underlying stone formation contribute to recurrent attacks. Especially in individuals with a history of stones at a young age, the risk of recurrence increases with familial predisposition and metabolic disorders (hypercalciuria, hyperuricosuria, hypocitraturia, etc.) [21]. In most studies conducted in the literature, inferences were made with limited data, fewer criteria, and fewer statistical methods. In this study, additional evaluation criteria were employed, resulting in a higher performance rate with the developed artificial intelligence model. The proposed model was tested on different findings and it was concluded that the developed model can be used in the detection of stone recurrence. These findings are consistent with previous studies, which have shown that artificial intelligence models can be effective not only in diagnosis processes but also in disease prevention and management [12,13]. However, our model has some limitations. We anticipate that future studies will increase the probability of success by training the model with datasets from different populations, thereby enhancing the sensitivity and specificity of the model. Additionally, integrating features such as genetic assays, metabolic assessments, and stone analysis into the model may increase predictive accuracy. Another limitation is the lack of cross-validation. While the stratified 80/20 split with a fixed random state supports repeatability, it may not fully capture variability across different splits. Future studies will evaluate cross-validation and test performance on independent external datasets to strengthen robustness and generalizability.

In conclusion, this study presents innovative solutions in the field of urology by highlighting the potential use of artificial intelligence in predicting the recurrence risk of kidney stones. It is envisioned that our model can serve as a clinical decision support tool for early diagnosis, individualized treatment plans, and disease prevention strategies. Testing our model with data collected from different centers is a separate advantage. Testing our model with data collected from more regions and different areas will further enhance the performance of our model, which is currently around 75%. However, additional validation studies are needed for the widespread adoption of the model in clinical practice. Steps to be taken in this direction can enable AI-based healthcare solutions to play a transformative role in kidney stone disease management.

## 5. Conclusions

The potential for urinary system stones recurring in terms of patients’ quality of life and the strain on the healthcare system stone disease is a serious issue. Consequently, it is crucial to create efficient techniques that can anticipate recurrence. In terms of tailored patient care, the use of preventative measures, and the elimination of needless diagnosis and follow-up procedures, early recurrence prediction can offer substantial benefits.

High recurrence prediction accuracy was achieved by the AI-based model created in this study using certain biomarkers and routine clinical data. The model’s results demonstrate that a comprehensive assessment of numerous factors influencing the likelihood of recurrence in stone disease can serve as an effective forecasting tool. Our research shows that incorporating these data-driven models into clinical decision support systems can improve the efficacy and personalization of patient follow-up while also improving diagnostic accuracy.

Both urology clinic patients and specialty doctors can benefit greatly from the created approach. By serving as a decision support system for professionals, it can help classify patient risks while giving patients the chance to start early information gathering and taking personal safety measures. By being included into cutting-edge health information systems, the model also has the potential to actively contribute to clinical research and public health initiatives.

## Figures and Tables

**Table 1:** Dataset description.

Part	Variable	Description	Unit
Part A	A1	Height	cm
	A2	Weight	kg
	A3	Waist Circumference	cm
	A4	Hip Circumference	cm
	A5	Age	years
Part B	B1	Gender	1: Male, 2: Female
	B2	Education Level	1: Literate, 2: Primary Education, 3: High School, 4: Bachelor’s Degree, 5: Master’s Degree
	B3	Diabetes	1: No, 2: Yes
	B4	Hypertension	1: No, 2: Yes
	B5	Cardiovascular Disease	1: No, 2: Yes
	B6	Inflammatory Bowel Disease	1: No, 2: Yes
	B7	History of Bowel Surgery	1: No, 2: Yes
	B8	Family History of Stone Disease	1: No, 2: Yes
	B9	Daily Water Consumption	L/day
	B10	Daily Physical Activity	0: Low, 1: Routine Daily Activities, 2: Regular Exercise
	B11	Tea Consumption	0: None, 1: 1–2 cups/day, 2: 4–5 cups/day, 3: >5 cups/day
	B12	Coffee Consumption	0: None, 1: 1–2 cups/day, 2: 4–5 cups/day, 3: >5 cups/day
	B13	Salt Intake	0: None, 1: Low, 2: Normal, 3: High
	B14	Animal Protein Intake	0: Low, 1: Normal, 2: High
	B15	Climate Type	0: Mild, 1: Cold, 2: Hot
	B16	Occupation Type	1: Active, 2: Sedentary
	B17	Medication for Stone Formation	0: No, 1: Yes
	B18	Sweating Level	0: None, 1: Little, 2: High
	B19	Smoking	0: No, 1: Yes
	B20	Alcohol Consumption	0: No, 1: Yes
Part C	C1	Body Mass Index	BMI
	C2	A Body Shape Index	ABSI
	C3	Body Roundness Index	BRI
	C4	Waist-to-Height Ratio	WHtR
	C5	Conicity Index	CI
	C6	Waist-to-Hip Ratio	WHR

**Table 2:** Recurrence/No Recurrence class-based performance comparison of classifiers.

Classifiers	Classes	Accuracy	Precision	Recall	F1-Score
XGBoost	Recurrence (−)	71.18	79.00	71.00	75.00
	Recurrence (+)	79.62	72.00	80.00	75.00
Cubic SVM	Recurrence (−)	72.88	67.00	73.00	70.00
	Recurrence (+)	61.11	67.00	61.00	64.00
Fine KNN	Recurrence (−)	69.49	72.00	69.00	71.00
	Recurrence (+)	70.37	68.00	70.00	69.00
DT	Recurrence (−)	71.18	75.00	71.00	73.00
	Recurrence (+)	74.07	70.00	74.00	72.00

**Table 3:** Performance comparison classifier without autoencoder and with autoencoder.

Model	Autoencoder	Accuracy	Precision	Recall	F1-Score
Cubic SVM	No	58.41	58.64	58.41	57.14
Fine KNN	No	68.14	68.00	62.96	65.38
DT	No	64.60	62.96	62.96	62.96
XGBoost	No	71.68	71.15	68.52	69.81
Cubic SVM	Yes	67.26	67.35	61.11	64.10
Fine KNN	Yes	69.91	67.86	70.37	69.11
DT	Yes	72.57	70.18	74.07	72.04
XGBoost	Yes	75.22	71.67	79.63	75.47

## Data Availability

Data used in this research are available upon request from the corresponding author.
